# CLDN18.2 expression and its impact on prognosis and the immune microenvironment in gastric cancer

**DOI:** 10.1186/s12876-023-02924-y

**Published:** 2023-08-16

**Authors:** Canming Wang, Yukai Wang, Jinxia Chen, Yi Wang, Chuhong Pang, Chen Liang, Li Yuan, Yubo Ma

**Affiliations:** 1 Zhejiang Cancer Hospital, Hangzhou Institute of Medicine (HIM), Chinese Academy of Sciences, Hangzhou, 310022 Zhejiang China; 2 The First Clinical Medical College of Zunyi Medical University, Zunyi, 563006 Guizhou China; 3 The Second Clinical Medical College of Zhejiang Chinese Medical University, Hangzhou, 310053 Zhejiang China

**Keywords:** Claudin 18.2, Gastric cancer, Prognosis, Immune microenvironment

## Abstract

**Background:**

The investigational use of zolbetuximab (IMAB362), a groundbreaking monoclonal antibody medication targeting claudin 18.2 (CLDN18.2), for treatment of advanced gastrointestinal cancers is currently underway. The unclear clinicopathological characteristics and tumour immune microenvironment of CLDN18.2-positive gastric cancer (GC) make it difficult to develop and optimize CLDN18.2-targeted therapies.

**Methods:**

A total of 451 tumour tissues, 342 matched paraneoplastic tissues, and 107 matched metastatic lymph nodes were collected from GC patients. These specimens were stained for CLDN18.2 expression and quantified using immunohistochemistry (IHC). Correlations between CLDN18.2 expression and clinicopathological features as well as immune-related factors were analysed. Survival curves were drawn using the Kaplan‒Meier approach, and independent factors affecting GC prognosis were identified using Cox regression analysis. Information from relevant databases was used for corroboration.

**Results:**

Expression of the CLDN18.2 gene was significantly lower in gastric tumour tissues than in normal tissues (*p* < 0.001) but comparable in metastatic lymph nodes (*p* = 0.851). CLDN18.2 expression was significantly associated with Borrmann type, degree of differentiation, PD-L1 expression, and survival in GC patients and was identified as an independent risk factor for patient prognosis (HR = 1.57, 95% CI 1.16–2.11, *p* = 0.003). There was no correlation between CLDN18.2 expression and HER2, Lauren type, tumour size, TNM stage, or any other clinicopathological characteristic. In CLDN18.2-positive tumours, fractions of CD4 + T cells and CD8 + T cells were significantly higher than those in CLDN18.2-negative tumours. Patients with CLDN18.2-negative expression and significant CD4 + T-cell or CD8 + T-cell infiltration had the best prognosis (5-year OS: 61.0%, *P* = 0.036; 5-year OS: 62.2%, *P* = 0.034).

**Conclusions:**

CLDN18.2 is expressed at a low level in tumour tissues and serves as an independent prognostic factor for patients with GC. Furthermore, CLDN18.2 correlates with immune infiltrating cells and PD-L1 expression.

**Supplementary Information:**

The online version contains supplementary material available at 10.1186/s12876-023-02924-y.

## Introduction

Gastric cancer (GC) is the fifth most common form of cancer worldwide and ranks as the fourth leading cause of cancer-related death [1]. The majority of GC cases are diagnosed at an advanced stage, which leads to poor prognosis for patients [2]. While several molecular targets have been investigated in clinical trials, only HER2-targeted trastuzumab and immune checkpoint inhibitors such as pembrolizumab or nivolumab have demonstrated effective response rates and been established as standard first-line therapies for advanced GC [3, 4]. There is an urgent need for development of new therapeutic targets and precise treatment regimens to improve the survival rate of patients with advanced GC.

Claudins (CLDNs) are a class of tight junction proteins that regulate the selective permeability and ion homeostasis of epithelial cells and mediate intercellular adhesion [5]. The human claudin gene family consists of 27 known members, each selectively facilitating passage of charged and uncharged solutes in a distinct manner. Claudin expression determines paracellular permeability, and expression of specific claudin family members varies across tissues. In the stomach, claudin-18 (CLDN18.2) is the most abundant form, whereas claudin-18.1 is predominantly expressed in the lung [6]. Cancer patients show claudin-18 deficiency (both pulmonary and gastric subtypes) early in the Correa cascade [7], and claudin-18 deficiency is an independent predictor of poor prognosis in cancer patients [8]. However, isoform 2 of claudin-18 (CLDN18.2), encoded by the CLDN18 gene, is typically inaccessible to antibodies due to its location within tight junctions of gastric mucosal cells. Nonetheless, during malignant transformation and loss of cell polarity, CLDN18.2 becomes exposed on the surface of tumour cells and is expressed in a significant proportion of primary gastric cancers and their metastases [9]. These unique features have drawn attention to the role of CLDN18.2 as a potential therapeutic target in GC.

Zolbetuximab is a first-in-class chimeric monoclonal IgG1 antibody that binds to CLDN18.2 and induces tumour cell death through antibody-dependent cytotoxicity (ADCC) and complement-dependent cytotoxicity (CDC) mechanisms [10–12]. A randomized phase II study (FAST) showed that in advanced gastric adenocarcinoma patients with CLDN18.2 expression, addition of zolbetuximab to first-line EOX therapy improved progression-free survival (PFS) and overall survival (OS) compared to EOX alone. Zolbetuximab plus EOX is generally well tolerated, with manageable adverse events [12]. Based on the therapeutic advantage observed in the overall patient population and in patients with moderate-to-strong CLDN18.2 expression in at least 70% of tumour cells, zolbetuximab 800/600 mg/m2 is being evaluated in phase III trials. In the MONO trial (phase IIa), zolbetuximab demonstrated a 9% objective response rate (ORR) and a 23% clinical benefit rate as second-line and subsequent first-line therapy for GC [13]. According to the latest results of a global phase III randomized trial (SPOTLIGHT) [14], zolbetuximab plus mFOLFOX6 significantly reduced patients’ risk of disease progression or death compared to mFOLFOX6 alone in CLDN18.2-positive and HER2-negative advanced gastric or gastroesophageal junction adenocarcinoma. Another global phase III trial, GLOW (NCT03653507), is currently comparing the therapeutic efficacy of CAPOX plus zolbetuximab versus CAPOX plus placebo in CLDN18.2-positive GC patients with recurrence or metastasis [15].

Recent retrospective studies have investigated the clinicopathological characteristics and prognostic implications of CLDN18.2-positive GC [16–19]. A Japanese retrospective study reported significantly higher CLDN18.2 expression in GCs of the diffuse histological subtype according to the Lauren classification and in high-grade (G3) tumours [19]. Another study by Jia K et al. explored the relationship between CLDN18.2 expression, clinicopathological characteristics, and immunotherapy outcomes in GC patients, finding that CLDN18.2-positive tumours had a significantly higher fraction of CD8 + T cells than CLDN18.2-negative tumours [20]. Tumour-infiltrating immune cells exhibit diverse phenotypes and functions, which can have both protumorigenic and antitumorigenic effects [21]. Understanding interactions among various components of the tumour microenvironment may provide insights into the biology of GC [22]. However, few studies have investigated the association between CLDN18.2 and HER2, PD-L1, and the tumour immune microenvironment in GC patients without prior preoperative anticancer treatment, including chemotherapy, radiotherapy, biotherapy, or immunotherapy.

The goal of this research was to explore expression of CLDN18.2 in gastric cancer, its clinical significance, and its relationship with the tumour immune microenvironment. In this study, we evaluated the expression level of CLDN18.2 in 451 tumour tissues, 342 paracancerous tissues, and 107 metastatic lymph nodes using immunohistochemistry (IHC). We also investigated independent factors influencing the prognosis of GC using Cox regression analysis, and we assessed TILs (CD3 + T cells, CD4 + T cells, and CD8 + T cells), HER2, PD-L1, and Foxp3 expression levels to explore their correlation with CLDN18.2. To support our findings, we incorporated information from several public medical databases.

## Materials and methods

### Patient samples

From March 2008 to August 2017, we selected a total of 451 gastric cancer (GC) patients admitted to the Cancer Hospital of Chinese Academy of Sciences (Zhejiang Cancer Hospital). The following inclusion criteria were used: (1) all of the samples had a pathological diagnosis of gastric cancer; (2) no antitumour treatment, such as chemoradiotherapy, biotherapy, or immunotherapy, had been administered before surgery; and (3) the patients’ medical records were complete. The exclusion criteria were as follows: (1) other types of malignant tumours; (2) metastasis from other malignant tumours; and (3) severe cardiopulmonary insufficiency, renal insufficiency, and other underlying diseases.

The study population consisted of 451 GC patients who underwent surgery at our centre. Haematoxylin and eosin (H&E)-stained sections were prepared for each patient’s surgically removed tumour. Paracancerous tissue was defined as tissue 2 cm from the edge of the tumour lesion, and in this study, the corresponding paracancerous tissue was obtained next to the tumour tissue. Metastatic lymph nodes were also obtained from these 451 gastric cancer patients. All available slides for the patients enrolled in this study were rereviewed following a standard histologic protocol by two pathology doctors holding the rank of Associate Chief Physician or above.

We collected a total of 451 tumour tissue samples, 342 paracancerous tissue samples, and 107 metastatic lymph node samples. The collected gastric tumour tissues, paracancerous tissues, and metastatic lymph nodes were treated with 4% paraformaldehyde and embedded in paraffin. Immunohistochemistry was performed on tissue microarrays prepared from these paraffin-embedded tissues to detect expression of CLDN18.2, Foxp3, HER2, and PD-L1 and CD3 + T cells, CD4 + T cells, and CD8 + T cells. Clinicopathological and survival data for the 451 patients were obtained through telephone follow-up and review of inpatient medical records. The collected data included patient age, sex, history of smoking, alcohol consumption, weight, family history of cancer, tumour location, Borrmann type, Lauren type, degree of differentiation, pathological type, tumour size, T stage, N stage, M stage, TNM stage, and tumour markers. TNM staging of gastric cancer was based on the eighth edition of the American Joint Committee on Cancer’s (AJCC) staging criteria.

### Immunohistochemical evaluation

After collection, the specimens of gastric tumour tissues, paracancerous tissues, and metastatic lymph nodes were fixed in formalin and embedded in paraffin. Two pathologists independently examined the samples, and representative samples of gastric tumour tissue, paracancerous tissue, and metastatic lymph nodes were selected for preparation of tissue microarrays. Among them, 2 tissue cores were taken from each case of gastric tumour tissue, 1 tissue core from each case of paracancerous tissue, and 1 tissue core from each case of metastatic lymph node. The tissue sections were dewaxed and rinsed with distilled water, followed by antigen retrieval. Subsequently, the tissue sections were rinsed three times for 5 min each with PBS. Immunohistochemistry was performed using specific antibodies against CLDN18.2, CD3 + T cells, CD4 + T cells, CD8 + T cells, Foxp3, HER2, and PD-L1. The antibodies used were against CLDN18.2 (Abcam, ab222512, dilution ratio 1:200), CD3 + T (Abcam, ab16669, dilution ratio 1:200), CD4 + T (Abcam, ab133616, dilution ratio 1:200), CD8 + T (Abcam, ab17147, dilution ratio 1:200), Foxp3 (Abcam, ab20034, dilution ratio 1:300), HER2 (Ventana, 790–4493), and PD-L1 (DAKO/Agilent, SK006, dilution ratio 1:50). The tissue microarrays were incubated overnight at 4 °C with the respective primary antibodies, followed by washing with PBS three times for 5 min each. Then, the appropriate secondary antibodies, i.e., goat anti-rabbit IgG H&L (PV-9003, ZSGB-BIO Corp., Shanghai, China; dilution ratio 1:1000) or goat anti-mouse IgG H&L (ISH-7003, ZSGB-BIO Corp., Shanghai, China; dilution ratio 1:500), were added. After incubation for 30 min, the slides were washed with PBS for 5 min three times. DAB colour development and haematoxylin staining of cell nuclei were performed using a DAB colour development kit (ZLI-9065, ZSGB-BIO Corp., Shanghai, China). Finally, the tissue microarrays were dehydrated and sealed with neutral gel closure (G8590, Solarbio, Beijing, China).

For detection of HER2 in gastric cancer, immunohistochemistry (IHC) was used as the preferred method based on the guidelines for detection of HER2 in gastric cancer from 2011 to 2016 [23]. Cases with IHC 3 + were classified as HER2 positive; patients with IHC 1 + and IHC 0 were classified as HER2 negative. Cases with IHC 2 + were considered “uncertain” and required further testing by in situ hybridization to confirm the HER2 status. If amplification was detected, the case was classified as HER2 positive, and if no amplification was detected, it was classified as HER2 negative.

Expression of PD-L1 was evaluated using the combined positive score (CPS), which is calculated by dividing the number of PD-L1-positive cells (including tumour cells, lymphocytes, and macrophages) by the total number of tumour cells. The CPS evaluation criteria involved the presence of membrane staining in tumour cells at any intensity, as well as membrane/cytoplasmic staining in lymphocytes and macrophages. The stained cells are expressed as a percentage of the total tumour cells, excluding necrotic cells, mesenchymal cells, carcinoma in situ, and other immune cells such as neutrophils, eosinophils, and plasma cells [24]. CPS was calculated as CPS= [number of PD-L1-positive cells (tumour cells, lymphocytes, macrophages)/total tumour cells] × 100. CPS ≥ 10 was scored as positive. The number of CD3 + T, CD4 + T and CD8 + T cells on the microarrays was counted. Patients were categorized into high and low expression groups based on the median number of stained cells.

Membrane expression of CLDN18.2 was assessed using the H-score system. The H-score was calculated using the Formula H-score = ∑ (IS × AP), where IS represents the staining intensity and AP represents the percentage of positively stained tumour cells. The staining intensity (IS) was assigned a value between 0 and 3 (0 = no staining, 1 = weak staining, 2 = intermediate staining, 3 = strong staining); the percentage of positively stained cells (AP) was assigned a value between 0 and 4 (0 = 0%, 1 = 1–25%, 2 = 26–50%, 3 = 51–75%, 4 = 76–100%). When assessing the percentage of positively stained cells, the cancer cells/epithelial cells in the normal adjacent tissue as well as the stromal compartment were included. The H-score ranges from 0 to 12, and an H-score of 1 or above indicates CLDN18.2-positive expression; an H-score of 0 indicates CLDN18.2-negative expression. Additionally, a cut-off value of H-score = 6 was defined to determine high expression (H-score ≥ 6) and low expression (H-score < 6) groups.

### Bioinformatics analysis

Expression of the CLDN18 gene in normal gastric tissue and gastric cancer was analysed using TNMplot.com (https://tnmplot.com/analysis/), which allows for online analysis of The Cancer Genome Atlas (TCGA), Genotype-Tissue Expression, and Gene Expression Omnibus (GEO) data [25].

The KM plotter tool was utilized to assess the effect of CLDN18 on survival in gastric cancer. KM plotter is based on databases from TCGA, GEO, and European Genome-phenome Archive (https://kmplot.com/analysis/). In addition, the Gene Expression Profiling Interactive Analysis (GEPIA) database, available at http://GEPIA.cancer-pku.cn, was used to analyse the impact of CLDN18 on survival in gastric cancer [26].

The TISIDB database (http://cis.hku.hk/TISIDB) was employed to examine relationships between CLDN18 expression and tumour-infiltrating lymphocytes (TILs), immunoinhibitors, and immunostimulators in gastric cancer [27]. Differential expression analysis was conducted using the TIMER database (https://cistrome.shinyapps.io/timer/) [28].

### Statistical analysis

Statistical analysis was conducted utilizing SPSS version 26.0 for Mac software (IBM Corp., Chicago, IL, USA). The chi-square and Fisher’s exact tests were utilized to compare categorical variables. One sample t test was used for comparison of two groups of continuous variables. One-way ANOVA was used for comparison of multiple continuous variables. The Kaplan‒Meier technique and the log-rank test were used to determine survival rates. We determined the hazard ratios (HRs) using Cox proportional hazards regression with 95% confidence intervals (CIs). Cancer mortality was designated as the endpoint. A *P* value < 0.05 was considered significant to include variables in Cox regression multivariate analysis. For each important variable, HRs and 95% CIs were calculated and compared to the reference category. A *P* value < 0.05 was considered significant. A forest plot was formulated using R 4.2.1 (The R Foundation for Statistical Computing, Vienna, Austria) with the forestmodel and survival packages.

## Results

### CLDN18.2 is expressed at low levels in gastric tumours and correlates negatively with the prognosis of patients

According to the analysis conducted at TNMplot.com, gene expression of CLDN18 in gastric tumours was significantly lower than that in normal tissues (Figure S1A, B, *P* < 0.01). However, when assessing the effect of CLDN18 expression on survival in gastric cancer patients using the GEPIA database and KM plotter, no significant correlation was found (Figure S1C, D, *P* > 0.05).

**Fig. 1:**
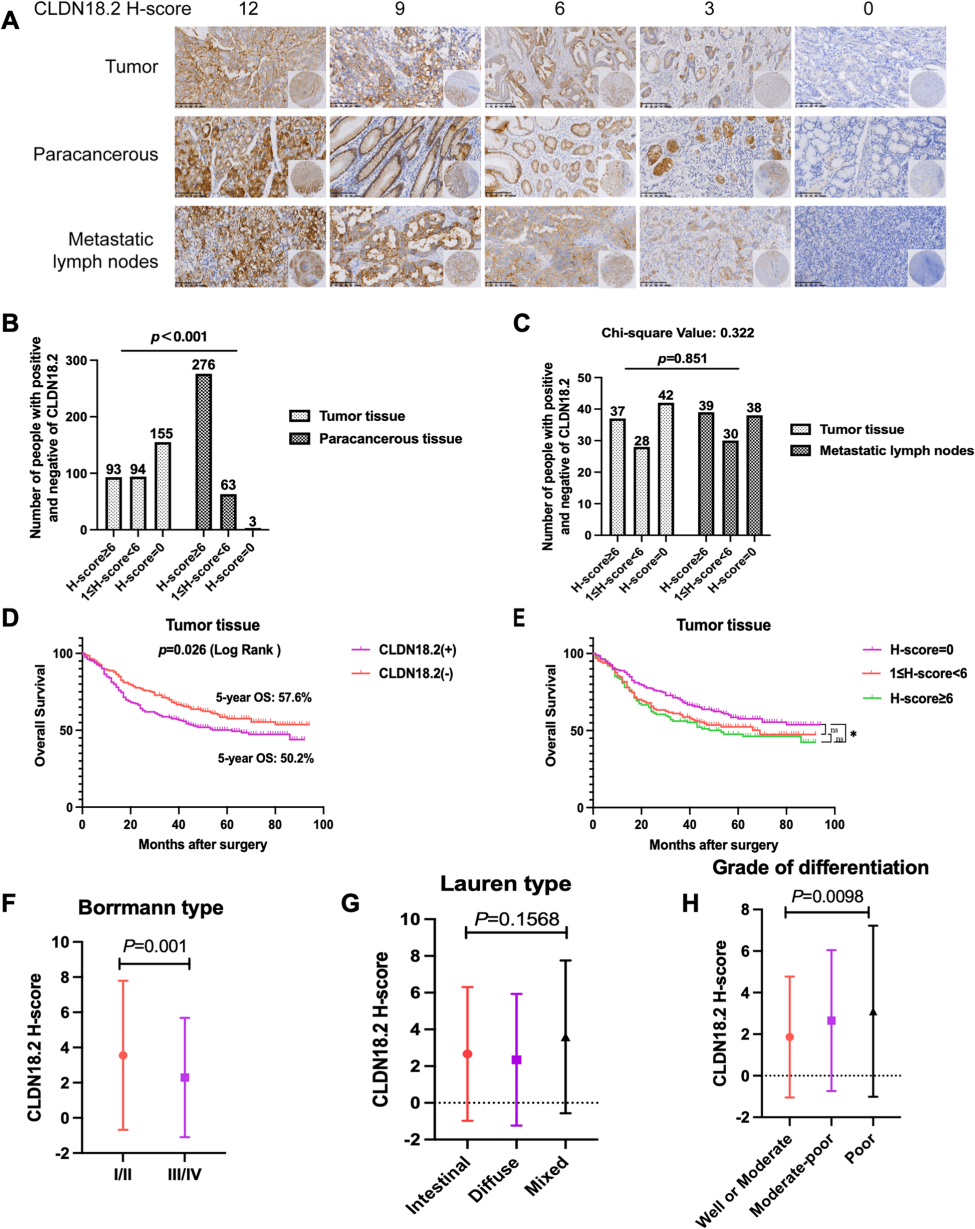
**A** Tissue microarrays of GC tumour tissues, paracancerous tissues and metastatic lymph nodes stained for the CLDN18.2 protein (4× and 20× magnifications). **B** Differential expression of CLDN18.2 in tumour and paracancerous tissues of GC. **C** Differential expression of CLDN18.2 in tumour tissues and metastatic lymph nodes of GC. **D**, **E** Kaplan‒Meier OS curves of GC patients with different CLDN18.2 levels in tumour tissues (log-rank test). * Statistically significant (*P* < 0.05). **F**-**H** Differences in CLDN18.2 expression levels by Borrmann type, Lauren type, and grade of differentiation

In this study, the cut-off value for CLDN18.2 expression was an H-score of 1. A total of 245 tumour samples (54.32%) showed positive expression of CLDN18.2; 206 samples were negative (Table 1). Representative images of IHC staining for CLDN18.2 are shown in Fig. 1A. Among the 342 matched patients, CLDN18.2 was positively expressed in 187 (54.68%) tumour tissues and 339 (99.12%) paracancerous tissues. High expression of CLDN18.2 (H-score ≥ 6) was significantly lower in tumour tissues than in paracancerous tissues (Fig. 1B and 27.19% vs. 80.7%, *p* < 0.001), indicating low expression of CLDN18.2 in gastric tumours. No significant differences in CLDN18.2 expression were found between gastric tumour tissues and matched metastatic lymph nodes (Fig. 1C, *p* = 0.851).

**Table 1:** Differential expression of CLDN18.2 in gastric cancer

Variables	N	H-score = 0	1 ≤ H-score < 6	H-score ≥ 6	Positive rate(≥1)
CLDN18.2	451	206	131	114	54.32%

Kaplan‒Meier survival curves were utilized to analyse the effect of CLDN18.2 on gastric cancer survival rates. Positive expression of CLDN18.2 was associated with shorter overall survival (OS) in GC patients (Fig. 1D, *P* = 0.026). However, patients with high levels of CLDN18.2 expression did not show a significantly worse prognosis than those with low levels of CLDN18.2 expression (Fig. 1E). These findings suggest a potential negative correlation between the level of CLDN18.2 expression in gastric tumour tissues and patient prognosis.

#### Relationship between expression of the CLDN18.2 protein and clinicopathological features

Based on Table 2, the expression level of CLDN18.2 in gastric cancer showed little significant correlation with various clinicopathological characteristics (chi-square and Fisher’s exact tests), except for the level of PD-L1 expression (*p* = 0.004). It is worth noting that this lack of significant correlation may be due to the grouping variable approach used in the analysis.

**Table 2:** Correlations between CLDN18.2 expression levels and the clinicopathological features of gastric cancer patients

Variables	CLDN18.2 expression		Total	Positive rate	χ2	*P value*
	Positive	Negative				
Age (year)						
≥ 65	117	95	212	55.2%	0.121	0.728
< 65	128	111	239	53.6%		
Sex						
Female	67	54	121	55.4%	0.073	0.787
Male	178	152	330	53.9%		
Family history (gastric cancer)						
Yes	25	16	41	61%	0.381	0.537
No	180	142	322	55.9%		
Smoking history						
Yes	55	53	108	50.9%	1.925	0.165
No	150	105	255	58.8%		
Drinking history						
Yes	43	35	78	55.1%	0.073	0.787
No	162	123	285	56.8%		
Weight loss						
Yes	59	47	106	55.7%	0.057	0.811
No	146	110	256	57.0%		
Tumour location						
Proximal stomach	84	59	143	58.7%	2.755	0.252
Distal stomach	149	140	289	51.6%		
Total stomach	11	6	17	64.7%		
Borrmann type						
I/II	81	48	129	62.8%	3.617	0.057
III/IV	120	109	229	52.4%		
Lauren type						
Intestinal	142	105	247	57.5%	5.642	0.060
Diffuse	66	74	140	47.1%		
Mixed	36	21	57	63.2%		
Tumour size (cm)						
≥ 5	137	119	256	53.5%	0.250	0.617
< 5	104	82	186	55.9%		
Grade of differentiation						
Well or Moderate	40	38	78	51.3%	3.369	0.186
Moderate-poor	84	51	135	62.2%		
Poor	115	100	215	53.5%		
Pathological type						
Adenocarcinoma	231	186	417	55.4%	2.562	0.109
Others	14	20	34	41.2%		
pT stage						
T1/2	19	24	43	44.2%	2.022	0.155
T3/4	220	176	396	55.6		
pN stage						
N0/1	73	71	144	50.7%	1.213	0.271
N2/3	166	129	295	56.3%		
pM stage						
M0	220	186	406	54.2%	0.141	0.707
M1	19	14	33	57.6%		
pTNM stage						
Ι/II	45	37	82	54.9%	0.008	0.930
III/IV	194	163	357	54.3%		
AFP (ng/ml)						
> 8.1	10	10	20	50%	0.432	0.511
≤ 8.1	180	133	313	57.5%		
CEA (ng/ml)						
> 5	48	38	86	57%	0.038	0.845
≤ 5	142	107	249	55.8%		
CA199 (U/ml)						
> 37	56	44	100	56%	0.030	0.863
≤ 37	134	101	235	57%		
CA724 (U/ml)						
> 6.9	26	29	55	47.3%	2.874	0.090
≤ 6.9	157	106	263	59.7%		
CA125 (U/ml)						
> 35	12	6	18	66.7%	0.635	0.425
≤ 35	161	121	282	57.1%		
CA50 (U/ml)						
> 25	26	17	43	60.5%	0.283	0.595
≤ 25	129	101	230	56.1%		
HER2						
Positive	17	24	41	41.5%	3.006	0.083
Negative	228	182	410	55.6%		
PD-L1						
Positive	74	38	112	66.1%	8.287	0.004*
Negative	171	168	339	50.4%		

a) * Statistically significant (*P* < 0.05)

To further investigate the relationship between CLDN18.2 expression levels and specific clinicopathological features, we analysed the association with Borrmann type (one sample t test), Lauren type (one-way ANOVA), and grade of differentiation (one-way ANOVA) (Fig. 1F-H). The analysis revealed that CLDN18.2 expression levels were higher in Borrmann types I and II than in Borrmann types III and IV (*p* = 0.001). Additionally, there was a strong correlation between the degree of gastric tumour hypodifferentiation and high CLDN18.2 expression levels (*p* = 0.0098). These findings suggest that CLDN18.2 expression levels are associated with the degree of tumour differentiation, Borrmann type, and PD-L1 expression levels.

#### CLDN18.2 is an independent prognostic marker for gastric cancer

Cox regression analysis was performed to explore factors outside of gastric cancer (GC) itself that might influence its prognosis. In univariate Cox analysis, positive CLDN18.2 expression was found to correlate significantly with overall survival (OS) (*P* = 0.028). Additionally, age, pT stage, pN stage, pTNM stage, tumour location, and tumour size were also identified as significant factors in univariate analysis (Table 3, all *P* < 0.05).

**Table 3:** Univariate analysis of prognostic factors for GC

Parameters	HR	95% CI	*P value*
Age (years) (≥ 65 vs. <65)	1.332	1.013–1.751	0.040*
Sex (male vs. female)	1.063	0.778–1.452	0.703
Tumour Location			< 0.001*
Proximal gastric cancer	1.373	1.026–1.837	0.033*
Distal gastric cancer	1		
Total stomach	3.379	1.902–6.004	< 0.001*
Borrmann type (III/IV vs. I/II)	1.340	0.960–1.870	0.080
Lauren type			0.063
Intestinal	1		
Diffuse	1.247	0.920–1.691	0.155
Mixed	1.570	1.052–2.344	0.027*
Tumour size (cm) (≥ 5 vs. <5)	1.708	1.273–2.293	< 0.001*
pT stage (T3/4 vs. T1/2)	2.966	1.520–5.788	0.001*
pN stage (N2/3 vs. N0/1)	3.038	2.101–4.392	< 0.001*
pTNM stage (III/IV vs. I/II)	2.299	1.464–3.612	< 0.001*
HER2	1.106	0.704–1.737	0.661
PD-L1	0.991	0.720–1.362	0.953
CLDN18.2 expression	1.366	1.035–1.803	0.028*

a) * Statistically significant (*P* < 0.05)

**Fig. 2:**
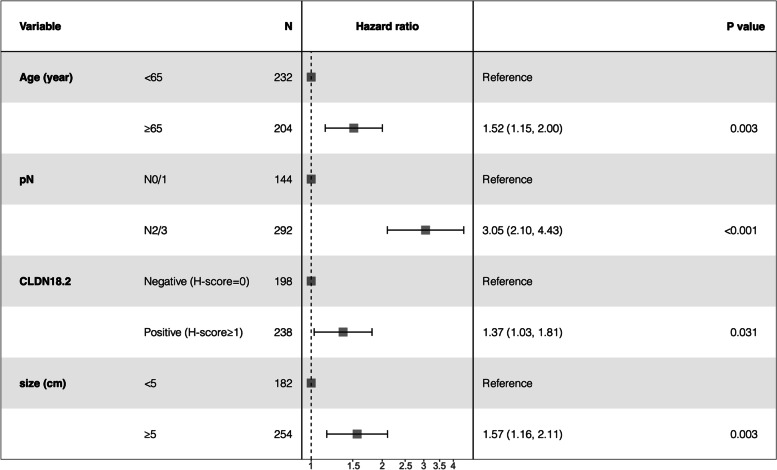
Forest plot of the results of the multivariate COX regression model for exploring potential risk factors for OS in GC patients from the Zhejiang Cancer Hospital

Multivariate Cox analysis was conducted to further examine the data that showed significance in univariate analysis. The results revealed significant associations between CLDN18.2 expression and OS (HR = 1.37, 95% CI = 1.03–1.81, *P* = 0.031), as well as age, pN stage, and tumour size (Fig. 2, all *P* < 0.05). These findings suggest that CLDN18.2 expression may act as an independent risk factor in gastric cancer.

#### CLDN18.2 expression regulates the GC immunological microenvironment

In our investigation of the immunological microenvironment in gastric cancer (GC) patients, we observed strong correlations between CLDN18.2 expression levels and CD4 + T cells, CD8 + T cells, and B cells according to the TISIDB database analysis (Figure S2A, all *p* < 0.05). However, when examining the TIMER database, we found that CLDN18.2 expression levels correlated with CD8 + T cells and Foxp3 expression levels (*p* < 0.05) but not with CD4 + T cells, B cells, CD274 (PD-L1) expression levels, or ERBB2 (HER2) expression levels (Figure S3, *p* > 0.05). These discrepancies between the two databases may contribute to the contradictions in the results.

**Fig. 3:**
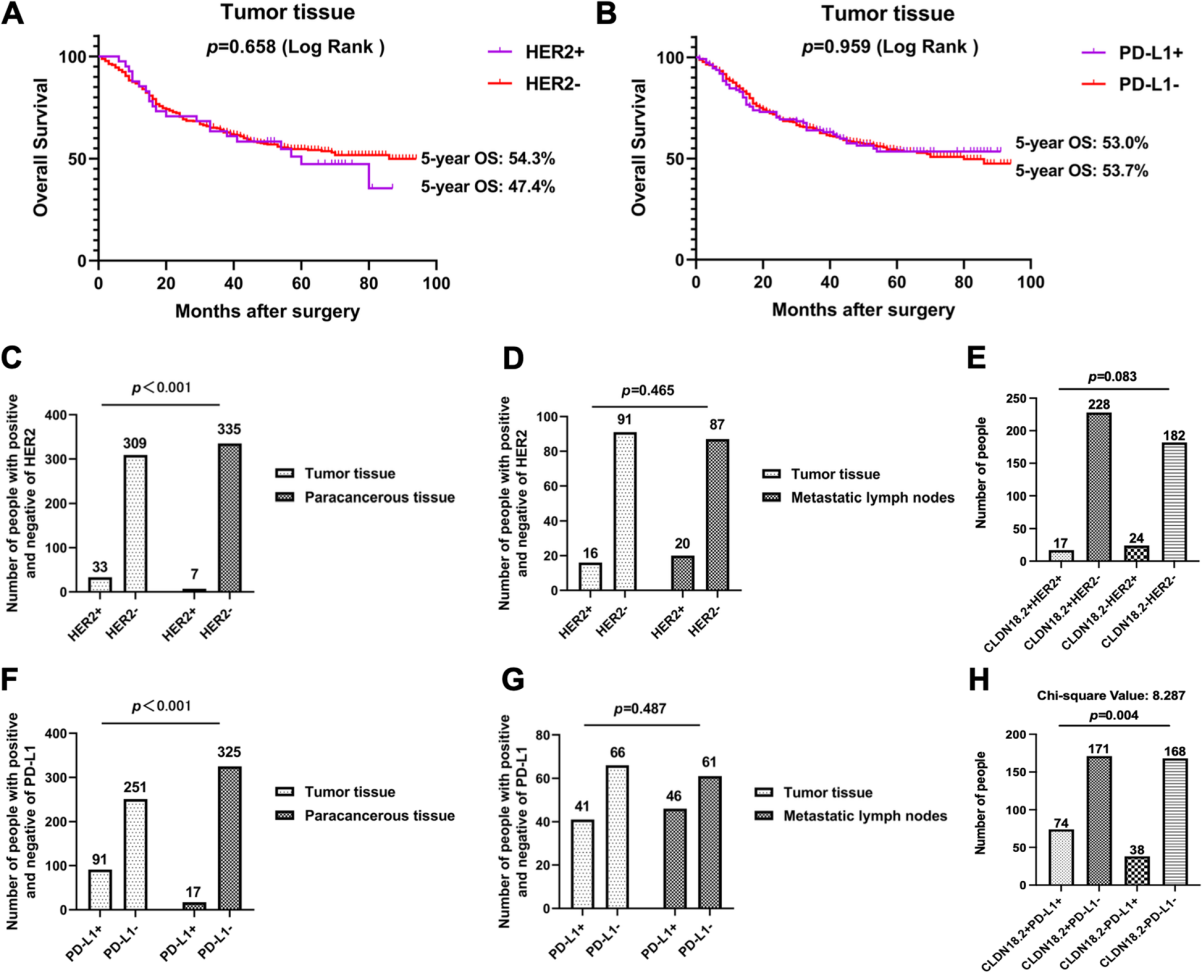
**A** Kaplan‒Meier OS curves of GC patients with different HER2 levels in tumour tissues (log-rank test). **B** Kaplan‒Meier OS curves of GC patients with different PD-L1 levels in tumour tissues (log-rank test). **C** Differential expression of HER2 in tumour and paracancerous tissues of GC. **D** Differential expression of HER2 in tumour tissues and metastatic lymph nodes of GC. **E** The number of samples was based on expression of CLDN18.2 and HER2 in tumour tissues. **F** Differential expression of PD-L1 in tumour and paracancerous tissues of GC. **G** Differential expression of PD-L1 in tumour tissues and metastatic lymph nodes of GC. **H** The number of samples was based on expression of CLDN18.2 and PD-L1 in tumour tissues

In our assessment of HER2 expression using the IHC method and PD-L1 expression using the combined positive score (CPS) in 451 GC samples, we found that neither HER2 nor PD-L1 expression levels significantly affected the overall survival (OS) of patients (Fig. 3A, B). The positive rate of HER2 and PD-L1 expression in tumour tissues was higher than that in paracancerous tissues (*p* < 0.05, Fig. 3C, F), but there was no significant difference compared to metastatic lymph nodes (Fig. 3D, G). We observed a positive correlation between CLDN18.2 expression levels and PD-L1 expression levels (*p* = 0.004, Fig. 3H), yet no significant correlation was found for HER2 expression levels (Fig. 3E).

**Fig. 4:**
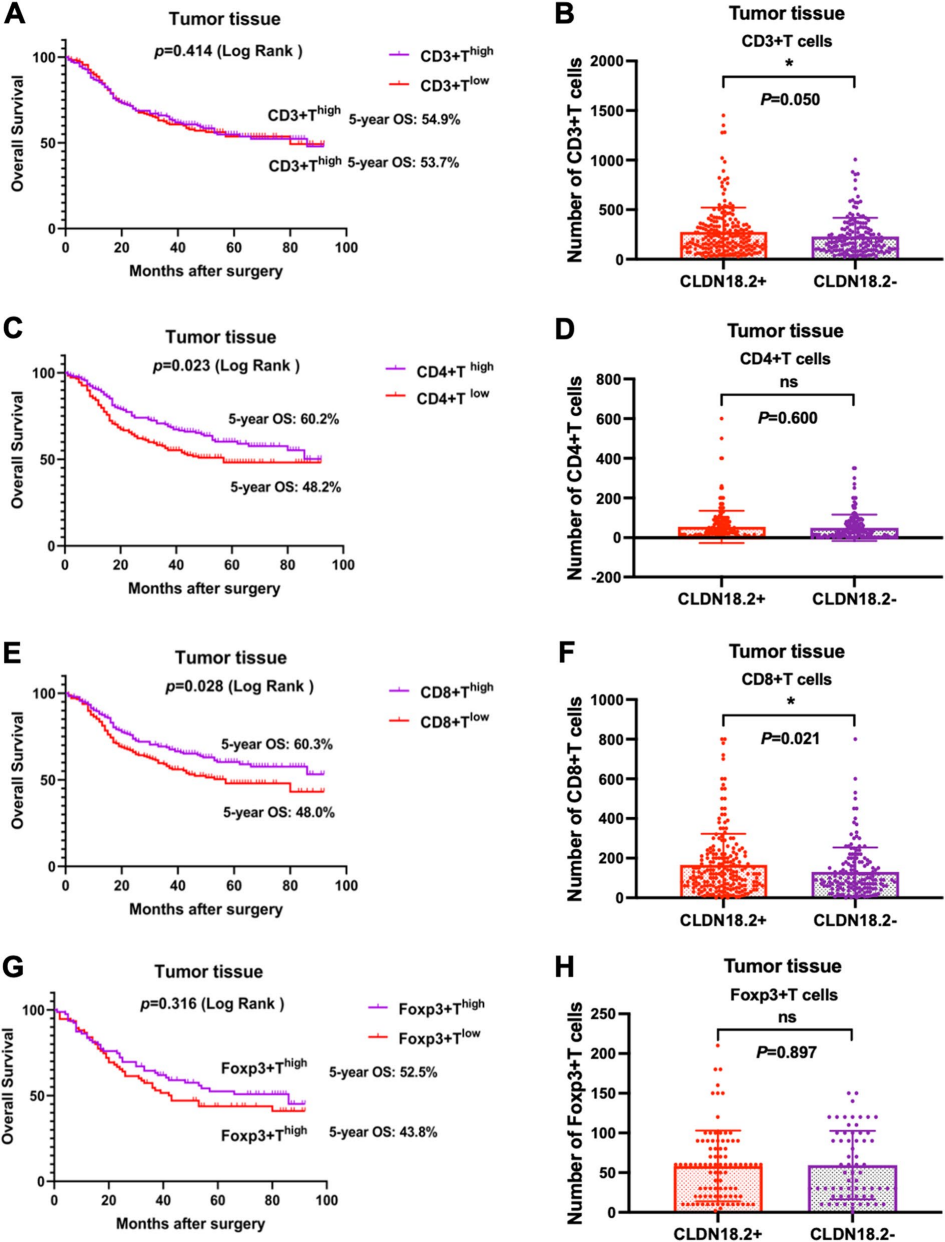
**A**, **C**, **E**, **G**) Kaplan‒Meier OS curves of GC patients with different CD3 + T (CD4 + T, CD8 + T and Foxp3) levels in tumour tissues (log-rank test). **B**, **D**, **F**, **H**) Association between CD3 + T (CD4 + T, CD8 + T and Foxp3) and CLDN18.2 expression in gastric cancer

In our evaluation of tumour-infiltrating lymphocytes (TILs) and Foxp3 expression in tumour tissues using immunohistochemistry (IHC) in 358 patients, patients with high levels of CD4 + T cells or CD8 + T cells exhibited better prognosis (Fig. 4C, E, all *p* < 0.05). However, levels of CD3 + T cells and Foxp3 expression had no significant effect on the prognosis of GC patients (Fig. 4A, G). Furthermore, we found that the number of CD3 + T cells and CD8 + T cells correlated positively with the level of CLDN18.2 expression (Fig. 4B, F) but not with CD4 + T cells and Foxp3 (Fig. 4D, H).

Additionally, we examined relationships between CLDN18.2 expression and immunosuppressive molecules (CD96, IL10RB, KDR, LGALS9, PVRL2, and VTCN1) as well as immune agonist molecules (C10orf54, ICOSLG, TNFRSF14, TNFRSF17, TNFSF13, and TNFSF15) using the TISIDB database (Figure S2B, C, all *p* < 0.05).

#### CLDN18.2- + CD4high status and CLDN18.2- + CD8high status predict better prognosis

**Fig. 5:**
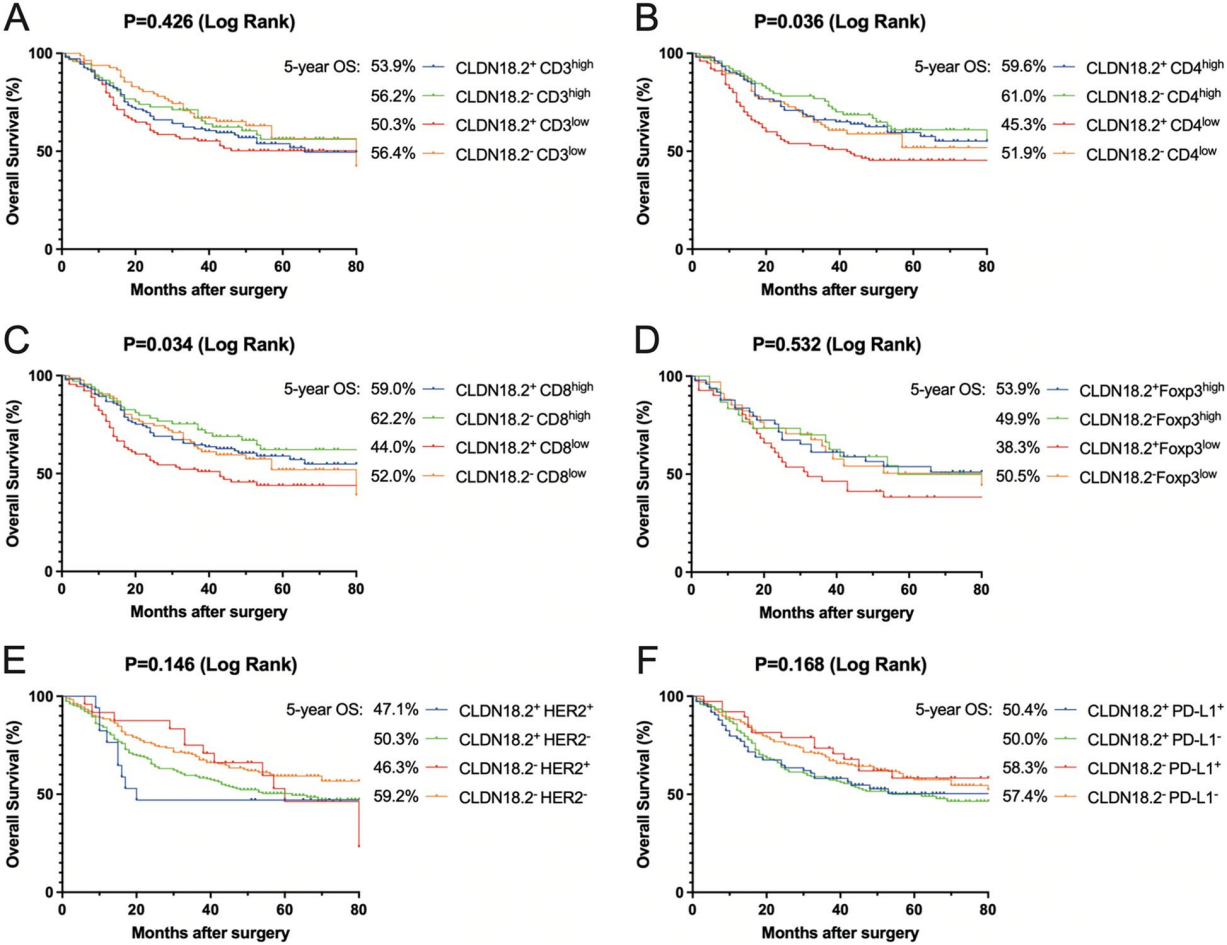
Kaplan–Meier survival curves for OS based on CLDN18.2 expression in tumour tissues in combination **A** with CD3 + T expression. **B** CD4 + T expression. **C** CD8 + T expression. **D** Foxp3 expression. **E** HER2 expression. **F** PD-L1 expression

We performed Kaplan‒Meier survival analysis in our investigation of the combined effects of CLDN18.2 with tumour-infiltrating lymphocytes (TILs), PD-L1, HER2, or Foxp3 on the prognosis of gastric cancer (GC) (Fig. 5). The analysis showed that the CLDN18.2^−^ + CD4^high^ group had the best prognosis, with a 5-year overall survival (OS) rate of 61.0%. In contrast, the CLDN18.2^+^ + CD4^low^ group had the worst prognosis, with a 5-year OS rate of 45.3% (Fig. 5A, *P* = 0.036). Prognostic value analysis of CLDN18.2 combined with CD8 + T cells showed similar results. The CLDN18.2^−^ + CD8^high^ group had the best prognosis among the four groups, with a 5-year OS rate of 62.2% (Fig. 5C, *p* = 0.034). On the other hand, combined analysis of CLDN18.2 with CD3 + T cells, HER2, PD-L1, or Foxp3 did not show statistically significant differences in prognosis (Fig. 5A, D-F, *p* > 0.05).

In summary, the combination of CLDN18.2-negative status with high levels of CD4 + T cells or CD8 + T cells predicts better prognosis in GC.

## Discussion

The prevalence of gastric cancer (GC) remains high worldwide, with over one million new cases diagnosed in 2020, and the disease has the fourth highest mortality rate among men and the fifth highest mortality rate among women of all malignancies [1]. Claudins, a family of proteins involved in forming tight junctions, play a crucial role in cell adhesion and are important targets for therapeutic interventions [6]. CLDN18.2, a specific subtype of claudin, is found in supramolecular complexes formed between normal epithelial cells and is maintained during malignant transformation, making it an attractive target for antibody binding and immune recruitment [9]. CLDN18.2 has the potential to be an important therapeutic molecule for patients with advanced GC, with a trend towards benefit in those who have higher CLDN18.2 expression [12]. The main objective of this study was to investigate differential expression of CLDN18.2 in gastric tumour tissues, adjacent normal tissues, and metastatic lymph nodes using immunohistochemistry (IHC) and then to explore the correlation of CLDN18.2 expression with various clinicopathological features, including survival. Additionally, we sought to investigate the relationship between CLDN18.2 and other factors, such as HER2, PD-L1, Foxp3, and tumour-infiltrating lymphocytes (TILs).

Previous studies have reported downregulation of CLDN18 in nearly half of GC cases, suggesting its potential as an early event in gastric carcinogenesis [7]. Studies involving CLDN18 knockout in mice revealed that its deletion leads to tumour formation, even in the absence of *Helicobacter pylori* infection [29]. Our analysis using the TNMplot.com database showed significantly lower expression of the CLDN18 gene in gastric tumour tissues than in normal tissues. In our cohort of 342 gastric tumour tissues and their corresponding adjacent tissues, we observed significantly lower positive expression of CLDN18.2 in tumour tissues (54.68%) than in adjacent tissues (99.12%, *p* < 0.001), which is consistent with database results. Several prior studies have also reported downregulation of CLDN18 as a characteristic of GC [8, 30, 31]. A low expression level of claudin proteins in tumour cells is consistent with the concept of destruction of tight junctions leading to disruption of epithelial cell cohesion and promoting cell invasiveness [32]. In addition, we did not find a significant difference in CLDN18.2 expression between gastric tumour tissues and corresponding metastatic lymph nodes.

In our patient cohort, positive expression of CLDN18.2 was associated with PD-L1 positivity, and lower CLDN18.2 expression was observed in Borrmann type 3/4 tumours and moderately or highly differentiated tumours [20]. These findings differ from a study by Kubota et al. [33], who reported an association between CLDN18.2 positivity and Borrmann type 4, KRAS amplification, low CD16 expression, and high CD68 expression. A related study reported that CLDN18.2 expression was associated with advanced stage (III, IV) [20]. Earlier single cohort studies demonstrated that expression of CLDN18.2 in GC is related to a diffuse phenotype [16, 19, 34]. However, in our study, we did not find differential expression of CLDN18.2 based on Lauren type or TNM stage. A recent meta-analysis also reported no significant correlation between CLDN18.2 expression and Lauren type or TNM stage [17]. The correlation of high CLDN18.2 expression with tumour hypodifferentiation may be one of the reasons for the poor prognosis of CLDN18.2-positive cancers.

In terms of prognosis, our study showed that patients with positive CLDN18.2 expression had significantly worse prognosis than those with negative expression (5-year overall survival: 50.2% vs. 57.6%, *p* = 0.026). However, we analysed the prognosis of GC patients in both databases using the median as the cut-off value, and CLDN18.2 expression did not show a significant difference in the prognosis of patients. Previous studies have also reported no correlation between CLDN18.2 expression and survival [17, 35]. Smaller studies by Sanada et al. (*n* = 65) and Jun et al. (*n* = 134) suggested that reduced CLDN18.2 expression is associated with poor prognosis [7, 8], which contradicts our findings. To further explore prognostic implications, we reclassified the CLDN18.2-positive expression group into high and low expression groups based on the median (H-score = 6) cut-off value., though there was no statistically significant difference in prognosis between these two groups.

CLDN18.2 has been identified as an independent factor affecting patient prognosis [6, 8], as confirmed by our univariate and multivariate analyses. However, contrary to the study by Jun KH et al. [8], positive CLDN18.2 expression in our study predicted poor prognosis for patients. This discrepancy may be attributed to differences in sample inclusion and exclusion criteria, as well as CLDN18.2 grouping differences. Notably, our patient sample had more advanced disease than the patients studied by Jun et al. (79.16% vs. 50.0% in stage III/IV). However, CLDN18 expression starts to decline in early gastric cancer or even in some intestinal chemosis of gastric tissue [7]. These reasons may have led to inconsistent results with previous studies.

The correlation between CLDN18.2 expression and other factors, such as HER2 and PD-L1, has been investigated in several studies. In advanced gastric or gastroesophageal junction cancers, Kubota Y et al. found no correlation between CLDN18.2 expression and HER2 or PD-L1 levels and that CLDN18.2 expression does not influence the efficacy of anti-PD-1 antibodies [33]. Similarly, Pellino A et al. reported no correlation between CLDN18 expression and HER2 or PD-L1 expression levels (CPS ≥ 1 and CPS ≥ 5) [36]. In our study, we used IHC to examine expression of CLDN18.2, HER2, and PD-L1 in 451 GC tissues and found a positive correlation between CLDN18.2 expression and PD-L1 (*p* = 0.004) but no correlation with HER2. We also observed high expression levels of HER2 and PD-L1 in gastric tumour tissues. Further investigation of the role of tumour-infiltrating lymphocytes (TILs) may contribute to a comprehensive understanding of the immune microenvironment and aid in the development of personalized immunotherapies [37]. TILs, including CD3+, CD4+, and CD8 + T cells, have been associated with a favourable prognosis in GC [38]. In our study, we found that high infiltration of CD4 + and CD8 + T cells was associated with better prognosis. Additionally, when CLDN18.2 expression was paired with CD4 + or CD8 + T-cell infiltration, patients with CLDN18.2-negative expression and significant T-cell infiltration had the best prognosis.

Our study has a few limitations. First, we included data from only a single medical institution, which is somewhat insufficient and may not be representative of the national patient population. Second, this was a retrospective study, which inevitably results in selection bias and information bias. Third, we used relatively old archival specimens for the study, which may have affected the rate of CLDN18.2 positivity as well as other indicators.

The findings suggest that CLDN18.2 may play a role in gastric cancer development and serve as a potential prognostic marker. It may also have a regulatory role in the immune microenvironment of gastric cancer. Further research is needed to elucidate the precise mechanisms and potential therapeutic implications of CLDN18.2 in gastric cancer.

## Conclusion

Expression of CLDN18.2 was found to be decreased in gastric tumour tissues compared to adjacent noncancerous tissues. Specifically, CLDN18.2 expression is significantly lower in Borrmann type 3/4 tumours, as well as in moderately or highly differentiated tumours. Additionally, a negative correlation exists between CLDN18.2 expression and patient prognosis. Interestingly, a combination of CLDN18.2-negative status with high levels of CD4 + T cells or CD8 + T cells is predictive of better prognosis in gastric cancer.

### Supplementary Information

**Additional file 1: Table S1.** Multivariate analysis of prognostic factors for GC. **Figure S1. **(A, B) Analysis of claudin18 expression in normal gastric tissue and gastric cancer using the TNMplot.com (https://tnmplot.com/analysis/) based on The Cancer Genome Atlas (TCGA), Genotype-Tissue Expression (GTEX), and Gene Expression Omnibus (GEO) databases. Significant differences by Mann-Whitney U test are marked with red*. (C) Assessment of the claudin18 effect on survival in gastric cancer using KM plotter (https://kmplot.com/analysis/) based on GEO, European Genome-phenome Archive, and TCGA databases. (D) Assessment of the claudin18 effect on survival in gastric cancer using the Gene Expression Profiling Interactive Analysis (GEPIA, http://GEPIA.cancer-pku.cn). HR: Hazard ratio. **Figure S2.** Correlations between CLDN18.2 expression and lymphocytes, immunoinhibitors, and immunostimulators in stomach adenocarcinoma (STAD). (A) Left panel: Correlation between CLDN18.2 expression and tumor-infiltrating lymphocytes (TILs) in pan-cancer. Right panel: Six TILs were positively correlated with CLDN18.2 expression in STAD. (B) Left panel: Correlation between CLDN18.2 expression and immunoinhibitors in pan-cancer. Right panel: Six Immunoinhibitors were positively correlated with CLDN18.2 expression in STAD. (C) Left panel: Correlation between CLDN18.2 expression and immunostimulators in pan-cancer. Right panel: Six immunostimulators were positively correlated with CLDN18.2 expression in STAD. **Figure S3.** (A) Correlation between CLDN18.2 expression and ERBB2 in gastric adenocarcinoma in the TIMER database. (B) Correlation between CLDN18.2 expression and CD274 in gastric adenocarcinoma in the TIMER database. (C) Correlation between CLDN18.2 expression and Foxp3 in gastric adenocarcinoma in the TIMER database. (D) Correlation between CLDN18.2 expression and immune cells in gastric adenocarcinoma in the TIMER database (https://cistrome.shinyapps.io/timer/).

## Data Availability

The datasets used and/or analysed during the current study are available from the corresponding author on reasonable request.

## References

[1] Sung H, Ferlay J, Siegel RL (2021). Global Cancer Statistics 2020: GLOBOCAN estimates of incidence and Mortality Worldwide for 36 cancers in 185 countries. CA Cancer J Clin.

[2] Song H, Zhu J, Lu D (2016). Molecular-targeted first-line therapy for advanced gastric cancer. Cochrane Database Syst Rev.

[3] Smyth EC, Nilsson M, Grabsch HI, van Grieken (2020). NC and Lordick F: gastric cancer. Lancet.

[4] Shitara K, Van Cutsem E, Bang YJ (2020). Efficacy and safety of Pembrolizumab or Pembrolizumab Plus Chemotherapy vs Chemotherapy alone for patients with First-line, Advanced Gastric Cancer: the KEYNOTE-062 phase 3 Randomized Clinical Trial. JAMA Oncol.

[5] Swisshelm K, Macek R, Kubbies M (2005). Role of claudins in tumorigenesis. Adv Drug Deliv Rev.

[6] Hagen SJ (2019). Unraveling a New Role for Claudins in gastric tumorigenesis. Cell Mol Gastroenterol Hepatol.

[7] Sanada Y, Oue N, Mitani Y, Yoshida K, Nakayama H, Yasui W (2006). Down-regulation of the claudin-18 gene, identified through serial analysis of gene expression data analysis, in gastric cancer with an intestinal phenotype. J Pathol.

[8] Jun KH, Kim JH, Jung JH, Choi HJ, Chin HM (2014). Expression of claudin-7 and loss of claudin-18 correlate with poor prognosis in gastric cancer. Int J Surg.

[9] Sahin U, Koslowski M, Dhaene K (2008). Claudin-18 splice variant 2 is a pan-cancer target suitable for therapeutic antibody development. Clin Cancer Res.

[10] Sahin U, Schuler M, Richly H (2018). A phase I dose-escalation study of IMAB362 (Zolbetuximab) in patients with advanced gastric and gastro-oesophageal junction cancer. Eur J Cancer.

[11] Tureci Ó¦, Mitnacht-Kraus R, Woll S, Yamada T, Sahin U (2019). Characterization of zolbetuximab in pancreatic cancer models. Oncoimmunology.

[12] Sahin U, Tureci O, Manikhas G (2021). FAST: a randomised phase II study of zolbetuximab (IMAB362) plus EOX versus EOX alone for first-line treatment of advanced CLDN18.2-positive gastric and gastro-oesophageal adenocarcinoma. Ann Oncol.

[13] Tureci O, Sahin U, Schulze-Bergkamen H (2019). A multicentre, phase IIa study of zolbetuximab as a single agent in patients with recurrent or refractory advanced adenocarcinoma of the stomach or lower oesophagus: the MONO study. Ann Oncol.

[14] Shitara K, Lordick F, Bang YJ (2023). Zolbetuximab plus mFOLFOX6 in patients with CLDN18.2-positive, HER2-negative, untreated, locally advanced unresectable or metastatic gastric or gastro-oesophageal junction adenocarcinoma (SPOTLIGHT): a multicentre, randomised, double-blind, phase 3 trial. Lancet.

[15] Ooki A, Yamaguchi K (2022). The dawn of precision medicine in diffuse-type gastric cancer. Ther Adv Med Oncol.

[16] Baek JH, Park DJ, Kim GY (2019). Clinical implications of Claudin18.2 expression in patients with gastric Cancer. Anticancer Res.

[17] Dottermusch M, Kruger S, Behrens HM, Halske C, Rocken C (2019). Expression of the potential therapeutic target claudin-18.2 is frequently decreased in gastric cancer: results from a large caucasian cohort study. Virchows Arch.

[18] Xu B, Liu F, Liu Q (2020). Highly expressed Claudin18.2 as a potential therapeutic target in advanced gastric signet-ring cell carcinoma (SRCC). J Gastrointest Oncol.

[19] Rohde C, Yamaguchi R, Mukhina S, Sahin U, Itoh K, Tureci O (2019). Comparison of Claudin 18.2 expression in primary tumors and lymph node metastases in japanese patients with gastric adenocarcinoma. Jpn J Clin Oncol.

[20] Jia K, Chen Y, Sun Y (2022). Multiplex immunohistochemistry defines the tumor immune microenvironment and immunotherapeutic outcome in CLDN18.2-positive gastric cancer. BMC Med.

[21] Zeng D, Li M, Zhou R (2019). Tumor Microenvironment characterization in gastric Cancer identifies prognostic and immunotherapeutically relevant Gene Signatures. Cancer Immunol Res.

[22] Kim ST, Cristescu R, Bass AJ (2018). Comprehensive molecular characterization of clinical responses to PD-1 inhibition in metastatic gastric cancer. Nat Med.

[23] Guideline Recommendations for HERDiGCG (2016). [Guidelines for HER2 detection in gastric cancer(2016)]. Zhonghua Bing Li Xue Za Zhi.

[24] Schoemig-Markiefka B, Eschbach J, Scheel AH (2021). Optimized PD-L1 scoring of gastric cancer. Gastric Cancer.

[25] Bartha A, Gyorffy B. TNMplot.com: a web Tool for the comparison of Gene expression in normal, Tumor and metastatic tissues. Int J Mol Sci. 2021;22(5):2622. 10.3390/ijms22052622 PMC7961455 33807717

[26] Tang Z, Li C, Kang B, Gao G, Li C, Zhang Z (2017). GEPIA: a web server for cancer and normal gene expression profiling and interactive analyses. Nucleic Acids Res.

[27] Ru B, Wong CN, Tong Y (2019). TISIDB: an integrated repository portal for tumor-immune system interactions. Bioinformatics.

[28] Li T, Fu J, Zeng Z (2020). TIMER2.0 for analysis of tumor-infiltrating immune cells. Nucleic Acids Res.

[29] Hagen SJ, Ang LH, Zheng Y (2018). Loss of tight Junction protein claudin 18 promotes progressive Neoplasia Development in mouse stomach. Gastroenterology.

[30] Zhang SJ, Feng JF, Wang L (2014). miR-1303 targets claudin-18 gene to modulate proliferation and invasion of gastric cancer cells. Dig Dis Sci.

[31] Matsuda Y, Semba S, Ueda J (2007). Gastric and intestinal claudin expression at the invasive front of gastric carcinoma. Cancer Sci.

[32] Yang L, Sun X, Meng X (2018). Differences in the expression profiles of claudin proteins in human gastric carcinoma compared with non–neoplastic mucosa. Mol Med Rep.

[33] Kubota Y, Kawazoe A, Mishima S (2023). Comprehensive clinical and molecular characterization of claudin 18.2 expression in advanced gastric or gastroesophageal junction cancer. ESMO Open.

[34] Coati I, Lotz G, Fanelli GN (2019). Claudin-18 expression in oesophagogastric adenocarcinomas: a tissue microarray study of 523 molecularly profiled cases. Br J Cancer.

[35] Dai J, Zheng H, Jin J, Cheng Y, Xu H. Claudin18.2 expression and clinicopathological features in cytology effusion specimens from gastric adenocarcinoma: a comparative study with tissue specimens. Cancer Cytopathol. 2023;131(6):365–72. 10.1002/cncy.22688 36793190

[36] Pellino A, Brignola S, Riello E et al. Association of CLDN18 protein expression with clinicopathological features and prognosis in Advanced Gastric and Gastroesophageal Junction Adenocarcinomas. J Pers Med. 2021;11:1095. 10.3390/jpm11111095 PMC8624955 34834447

[37] Chen Y, Jia K, Sun Y (2022). Predicting response to immunotherapy in gastric cancer via multi-dimensional analyses of the tumour immune microenvironment. Nat Commun.

[38] Zhang D, He W, Wu C (2019). Scoring system for Tumor-Infiltrating lymphocytes and its prognostic value for gastric Cancer. Front Immunol.

