# Supplementary material for: CLDN18.2 expression and its impact on prognosis and the immune microenvironment in gastric cancer
Source: BMC Gastroenterol. 2023 Aug 16;23:283. doi: 10.1186/s12876-023-02924-y (PMC10428652; doi:10.1186/s12876-023-02924-y)
Supplement: Supplementary file 1 — Additional file 1: Table S1. Multivariate analysis of prognostic factors for GC. Figure S1. (A, B) Analysis of claudin18 expression in normal gastric tissue and gastric cancer using the TNMplot.com (https://tnmplot.com/analysis/) based on The Cancer Genome Atlas (TCGA), Genotype-Tissue Expression (GTEX), and Gene Expression Omnibus (GEO) databases. Significant differences by Mann-Whitney U test are marked with red*. (C) Assessment of the claudin18 effect on survival in gastric cancer using KM plotter (https://kmplot.com/analysis/) based on GEO, European Genome-phenome Archive, and TCGA databases. (D) Assessment of the claudin18 effect on survival in gastric cancer using the Gene Expression Profiling Interactive Analysis (GEPIA, http://GEPIA.cancer-pku.cn). HR: Hazard ratio. Figure S2. Correlations between CLDN18.2 expression and lymphocytes, immunoinhibitors, and immunostimulators in stomach adenocarcinoma (STAD). (A) Left panel: Correlation between CLDN18.2 expression and tumor-infiltrating lymphocytes (TILs) in pan-cancer. Right panel: Six TILs were positively correlated with CLDN18.2 expression in STAD. (B) Left panel: Correlation between CLDN18.2 expression and immunoinhibitors in pan-cancer. Right panel: Six Immunoinhibitors were positively correlated with CLDN18.2 expression in STAD. (C) Left panel: Correlation between CLDN18.2 expression and immunostimulators in pan-cancer. Right panel: Six immunostimulators were positively correlated with CLDN18.2 expression in STAD. Figure S3. (A) Correlation between CLDN18.2 expression and ERBB2 in gastric adenocarcinoma in the TIMER database. (B) Correlation between CLDN18.2 expression and CD274 in gastric adenocarcinoma in the TIMER database. (C) Correlation between CLDN18.2 expression and Foxp3 in gastric adenocarcinoma in the TIMER database. (D) Correlation between CLDN18.2 expression and immune cells in gastric adenocarcinoma in the TIMER database (https://cistrome.shinyapps.io/timer/). [file 12876_2023_2924_MOESM1_ESM.docx]

**Supplementary Tables**

Table S1 Multivariate analysis of prognostic factors for GC.

| Parameters | HR | 95%CI | *P value* |
| --- | --- | --- | --- |
| Age (years) (≥65 vs. <65) | 1.52 | 1.15-2.00 | **0.003*** |
| Tumour size (cm) (≥5 vs. <5) | 1.57 | 1.16-2.11 | **0.003*** |
| pN stage (N2/3 vs. N0/1) | 3.05 | 2.10-4.43 | **<0.001*** |
| CLDN18.2 expression | 1.37 | 1.03-1.81 | **0.031*** |

1. * Statistically significant (***P*** < 0.05)

**Supplementary Figures**


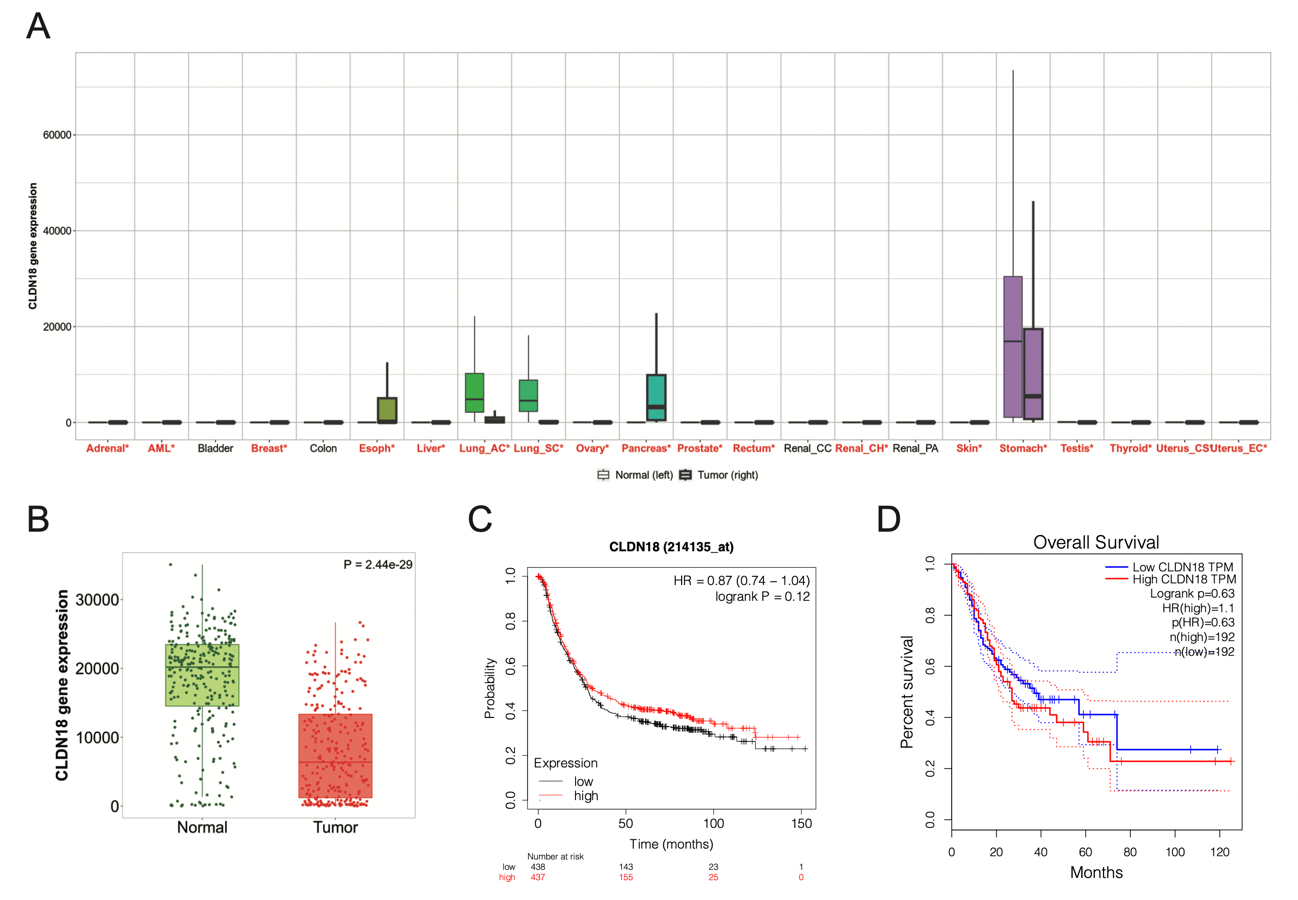


Figure S1


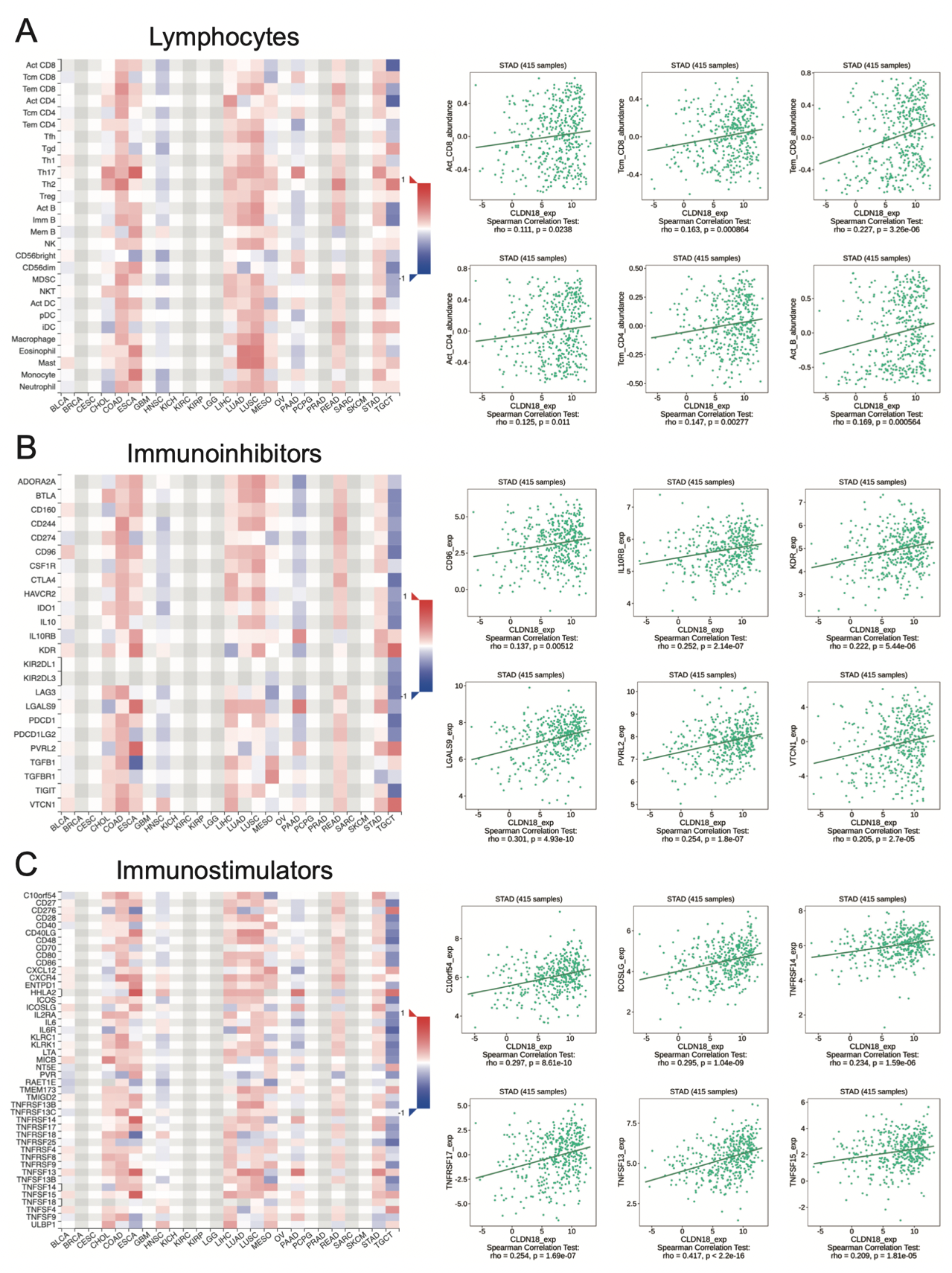
.

Figure S2


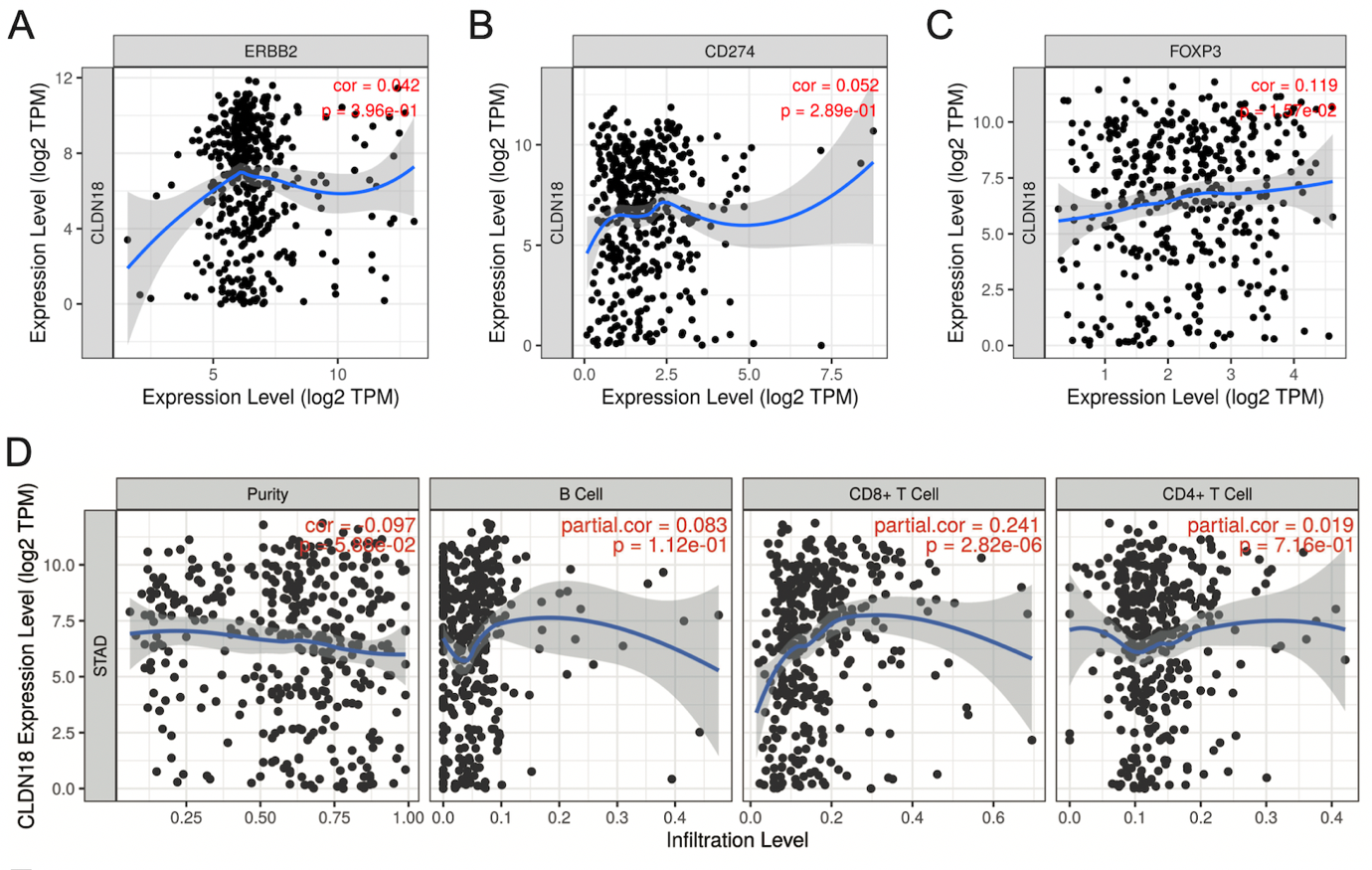


Figure S3
